# Gerstmann-Sträussler-Scheinker disease revisited: accumulation of covalently-linked multimers of internal prion protein fragments

**DOI:** 10.1186/s40478-019-0734-2

**Published:** 2019-05-29

**Authors:** Laura Cracco, Xiangzhu Xiao, Satish K. Nemani, Jody Lavrich, Ignazio Cali, Bernardino Ghetti, Silvio Notari, Witold K. Surewicz, Pierluigi Gambetti

**Affiliations:** 1 Department of Pathology, Case Western Reserve University, Cleveland, OH USA; 2 Department of Physiology and Biophysics, Case Western Reserve University, Cleveland, OH USA; 3 National Prion Disease Pathology Surveillance Center, Case Western Reserve University, Cleveland, OH USA; 4 Department of Pathology and Laboratory Medicine, Indiana University School of Medicine, Indianapolis, IN USA

**Keywords:** Creutzfeldt-Jakob disease, Prion protein, Aggregate formation, Multimers, Mass spectrometry, Epitope mapping

## Abstract

Despite their phenotypic heterogeneity, most human prion diseases belong to two broadly defined groups: Creutzfeldt-Jakob disease (CJD) and Gerstmann-Sträussler-Scheinker disease (GSS). While the structural characteristics of the disease-related proteinase K-resistant prion protein (resPrP^D^) associated with the CJD group are fairly well established, many features of GSS-associated resPrP^D^ are unclear. Electrophoretic profiles of resPrP^D^ associated with GSS variants typically show 6–8 kDa bands corresponding to the internal PrP fragments as well as a variable number of higher molecular weight bands, the molecular nature of which has not been investigated. Here we have performed systematic studies of purified resPrP^D^ species extracted from GSS cases with the A117V (GSS^A117V^) and F198S (GSS^F198S^) PrP gene mutations. The combined analysis based on epitope mapping, deglycosylation treatment and direct amino acid sequencing by mass spectrometry provided a conclusive evidence that high molecular weight resPrP^D^ species seen in electrophoretic profiles represent covalently-linked multimers of the internal ~ 7 and ~ 8 kDa fragments. This finding reveals a mechanism of resPrP^D^ aggregate formation that has not been previously established in prion diseases.

**Electronic supplementary material:**

The online version of this article (10.1186/s40478-019-0734-2) contains supplementary material, which is available to authorized users.

## Introduction

A well-known feature of human prion diseases is the presence of three distinct etiologic forms - sporadic, inherited and acquired by infection. A further complicating factor is the great phenotypic variability within each of these etiologically distinct groups. For example, the sporadic form alone encompasses seven major disease phenotypes [15, 16, 39], and many different variants have been reported for inherited prion diseases [20, 21]. This phenotypic variability is believed to be directly related to (and likely encoded in) distinct strains of the disease-related prion protein (PrP^D^). However, the nature and extent of specific structural differences between phenotype-specific PrP^D^ strains remain poorly understood [15].

According to the classification based on electrophoretic profiles of the proteinase K (PK)-resistant PrP^D^ (resPrP^D^), most cases of sporadic and inherited prion diseases fall into two broadly defined groups: Creutzfeldt-Jakob disease (CJD) and Gerstmann-Sträussler-Scheinker disease (GSS). CJD is characterized by the presence of relatively large resPrP^D^ fragments which, depending on the N-terminus, are classified as type 1 (typically starting at residue G82) and type 2 (starting at residue S97) [15, 26]. Both fragment types extend to the C-terminus and include the glycosylphosphatidylinositol (GPI) anchor [16, 39]. Electrophoretic profiles of these fragments include bands of approximately 30 and 27 kDa (representing the di- and mono-glycosylated forms) as well as bands of 21 and 19 kDa which represent the un-glycosylated form in resPrP^D^ types 1 and 2, respectively. Collectively, these three resPrP^D^ fragments are commonly referred to as PrP 27–30 [8]. A remarkably different electrophoretic profile is observed in GSS, where the most prominent and by far best characterized resPrP^D^ species is a 6–8 kDa fragment encompassing internal residues from within the ~ 70–150 region [12, 25, 27, 33, 34]. Higher molecular weight (hmw) bands of variable estimated molecular weights have also been reported, occasionally prompting the hypothesis that they represent multimers. However, the molecular nature of the PrP fragments giving rise to these bands remains enigmatic [12, 17, 23].

To bridge this gap, here we have performed detailed analysis of purified resPrP^D^ preparations from GSS cases harboring the A117V (GSS^A117V^) and F198S (GSS^F198S^) PrP mutations. Our data demonstrate that high molecular weight species seen in electrophoretic profiles of GSS^A117V^ and GSS^F198S^ resPrP^D^ represent covalently-linked multimers of the same internal PrP fragments that are present (as monomers) in the ~ 7 and ~ 8 kDa bands.

## Materials and methods

### Reagents and antibodies

β–mercaptoethanol, Dithiothreitol, Laemmli Sample Buffer, Non-fat dry milk, Sodium dodecyl sulfate (SDS), Tris Buffered Saline (TBS), Tris/Glycine/SDS buffer, Tris/Glycine Buffer, Tween 20 and 15% Criterion Tris-HCl polyacrylamide precast gels were purchased from Bio-Rad Laboratories (Hercules, CA, USA). Benzonase, Calcium chloride, Complete Ultra Protease Inhibitor Cocktail Tablets, Dulbecco’s PBS (D-PBS), Kodak Biomax MR and XAR films, NaCl, Nonidet P-40, N-Lauroylsarcosine sodium salt solution (Sarkosyl), Phenylmethanesulfonyl fluoride (PMSF), Polyvinylidene difluoride (PVDF) membrane (Immobilon-P), Proteinase K, Sodium deoxycholate and Tris-HCl came from MilliporeSigma (Burlington, MA, USA) whereas Ethylenediaminetetraacetic acid (EDTA) from Promega (Madison, WI, USA). Glycerol-free PNGase F was from New England Biolabs Inc. (Ipswich, MA, USA); Acetonitrile, Colloidal Blue Staining Kit, Ethanol, Formic Acid, 8 M Guanidine-HCl Solution, Methanol, Pierce ECL 2 Western Blotting Substrate and Trypsin from Thermo Fisher Scientific Inc. (Waltham, MA, USA).

The following antibodies (Abs) were used in the study: 8B4 (to human PrP residues 36–43) [22], SAF32 (to human octarepeat region) [14] (Cayman Chemical, Ann Arbor, MI, USA), 3F4 (to human PrP residues 106–110) [19, 42], F89 (to human PrP residues 139–142) (Thermo Fisher Scientific Inc., Waltham, MA, USA), L42 (to human PrP residues 145–150) [18, 38] (R-Biopharm, AG, Darmstadt, Germany) and SAF70 (to human PrP residues 156–162) [14]. Secondary Ab was sheep anti-mouse IgG, HRP-linked whole antibody (GE Healthcare Life Sciences, Chicago, IL, USA).

### Brain tissues

All frozen brain tissues were obtained from the National Prion Disease Pathology Surveillance Center (NPDPSC). Frontal cortex samples from two cases each of GSS A117V–129V (129MV and 129VV) and F198S-129V (129MV) were used. All cases were used for immunoblotting, but only one case of GSS^A117V^ and GSS^F198S^, both with 129MV genotype, were used for mass spectrometry. A case of sCJDMV1 was used as control.

### Preparation of brain homogenates

Brain homogenates (BH) were prepared as previously described [13].

### Proteinase K digestion

Samples were incubated for 1 h at 37 °C with different amounts of PK, as follows: GSS^A117V^: purified PrP^D^ (PK 2 U/ml); GSS^F198S^: BH (PK 5 U/ml), purified PrP^D^ (PK 10 U/ml); sCJDMV1: purified PrP^D^ (PK 10 U/ml). The reaction was stopped by the addition of 3 mM PMSF.

### Epitope mapping

The optimal PK concentrations and the volumes of purified GSS^F198S^, GSS^A117V^ and sCJDMV1 resPrP^D^ loaded into the gel were selected to obtain a clear visibility of all the bands with 3F4. Recombinant human PrP full-length (23–231) and truncated (90–231) species were used as molecular weight markers.

### Methanol/ethanol precipitation

Methanol precipitation was performed as previously described [13]. For ethanol precipitation the same procedure was followed with the exception of 9 volumes of pre-chilled ethanol instead of 5 volumes used to dilute the sample.

### PrP deglycosylation

Samples were treated with glycerol-free PNGase F in accordance with manufacturer’s instructions.

### Electrophoresis and immunoblot

Protein samples were run on 15% Criterion Tris-HCl polyacrylamide precast gels and then subjected to immunoblot (60 V for 2 h) using Immobilon-P PVDF membranes. After 1 h blocking in 5% non-fat dry milk in TBS with 0.1% Tween 20 (TBS-T), the membranes were incubated with primary Ab overnight at 4 °C; they were then washed with TBS-T, incubated for 1 h at RT with the secondary HRP-conjugated Ab and then washed again prior to developing by enhanced chemiluminescence reaction using ECL 2 western blotting substrate, as per manufacturer’s instructions. Kodak MR and XAR films were used to capture the signal.

### ResPrP^D^ purification

ResPrP^D^ purification was performed as reported in Bolton et al. [7] and adapted by Zou et al. [40]. Rotor SW55Ti (Beckman Coulter, Brea, CA, USA) was used for ultracentrifugation.

### In-gel trypsin digestion of purified resPrP^D^

In-gel trypsin digestion was performed according to an established protocol [31]. Briefly, the protein bands of interest (visualized by Colloidal Coomassie blue staining) were excised from the gel, cut into small cubes and destained. The destained gel pieces were then dehydrated and followed by reduction/alkylation step. For digestion, trypsin solution (15 ng/μl) was added and gel pieces were incubated overnight at 37 °C with shaking. The supernatant was then transferred into a new Eppendorf tube and the remaining peptides in gel pieces were extracted, supernatants were pooled, concentrated and stored at − 80 °C until analyzed by Nano LC-MS.

### Nano LC-MS/MS

Nanospray LC-MS-MS analysis was performed using an LTQ Orbitrap XL mass spectrometer equipped with nanoelectrospray source (Thermo Scientific, San Jose, CA, USA). Trypsin-digested samples were loaded onto a C-18 trap column (to remove salts) and separated on a C-18 column connected to an emitter. Separation was performed using a Dionex UltiMate 3000 system (Thermo Scientific, San Jose, CA, USA) and a gradient of acetonitrile in water containing 0.1% formic acid. The flow rate was 300 nl/min. The mass spectrometer was externally calibrated using a Pierce LTQ ESI positive ion calibration solution (Thermo Scientific, catalog number 88322). Full scan experiments were acquired in the m/z 300–1800 range at a resolution of 30,000 (FWHM at m/z 400). The following source settings were used: spray voltage = 4.2 kV; capillary temperature = 200 °C. Data-dependent MS^n^ (*n* = 2) were acquired at ITMS using collision induced dissociation (CID); the top 14 intense ions were subjected for further fragmentation. Calculation of elemental formulae was performed on the mono-isotopic peak of each ion cluster using Xcalibur software v2.2 with a mass tolerance of 3 to 5 ppm. MS/MS raw files were searched using MASCOT Deamon engine against the database containing sequence of human prion protein 129M/V with mutation A117V or F198S. Trypsin/P search parameters for Mascot peptide identification included one missed tryptic cleavage, fixed carbamidomethylation (+ 57 Da, Cys), and variable oxidation (+ 16 Da, Met). Mass tolerances of 2.0 and 1.0 Da were used for parent and monoisotopic fragment ions, respectively. The resulting files generated by MASCOT were used for peptide identification with the constraints that only MASCOT ion scores greater than 10 were considered. The percentage of 129M and 129V PrP in resPrP^D^ samples was calculated by the spectral counting method [4].

## Results

**Fig. 1:**
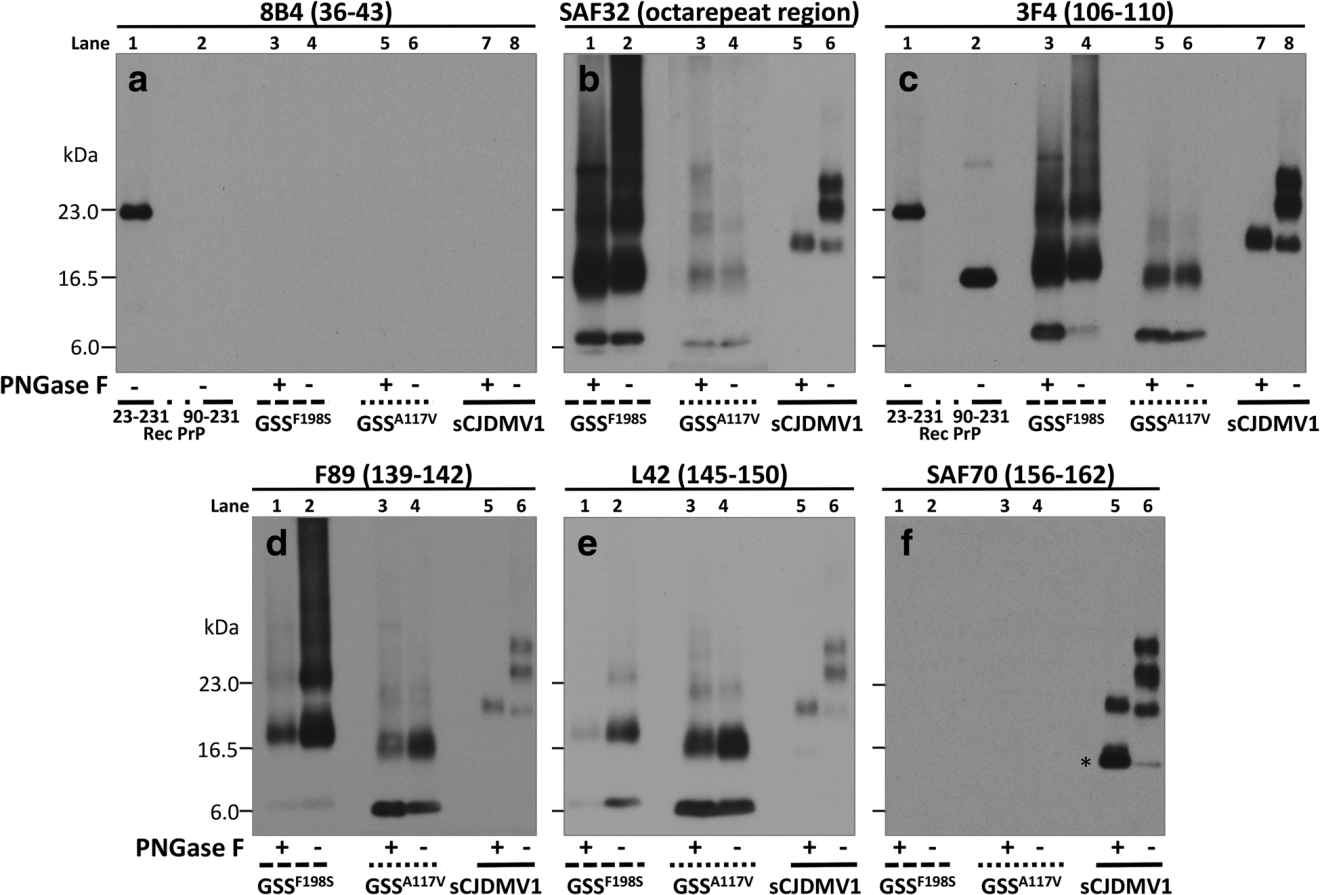
Epitope mapping combined with deglycosylation of purified resPrP^D^ associated with GSS^F198S^, GSS^A117V^ and sCJDMV1 used as control. PNGase F-treated or untreated, PK-resistant PrP^D^ (resPrP^D^) purified (see Materials and Methods) from GSS^F198S^, GSS^A117V^ and sCJDMV1 control were blotted and probed with the Abs to PrP denoted, with their epitopes, at the top of each panel. Panel **a**: Only full-length (PK-untreated) recombinant PrP (23–231) was detected by this Ab to the proximal N-terminal region confirming the absence of resPrP^D^ with complete N-terminus. Panels **b** to **e**: Both GSS^F198S^ and GSS^A117V^ resPrP^D^ conformers were selectively detected by the same Abs to PrP N- and C-terminal regions; both also showed no major variation of the resPrP^D^ banding pattern following deglycosylation indicating that most of the resPrP^D^ is unglycosylated in both conditions. Although overall similar, the two resPrP^D^ profiles differed especially in the higher molecular weight region suggesting a distinct repertoire, or relative proportions, of polymers in the two GSS variants. The GSS banding patterns clearly differed from the three-band pattern of sCJDMV1, two of which are glycosylated (See Results for detailed description). Panel **c** includes the bands of both 23–231 and 90–231 recombinant PrP which have been used as molecular weight markers. In **b** the portion of the panel with the GSS^A117V^ samples required longer exposure. In panel **f**, * indicates the 13 kDa component of the glycosylated and anchor bearing PrP 12/13 C-terminus fragments with N-termini at residues 162–167 and 154–156, respectively [24, 41]

The characteristics of resPrP^D^ in GSS^F198S^ and GSS^A117V^ were examined and compared with those of sCJDMV1 resPrP^D^, used as control, by combining epitope mapping with enzymatic deglycosylation on immunoblots of purified resPrP^D^ preparations (Fig. 1). Overall, GSS^F198S^ and GSS^A117V^ showed similar electrophoretic profiles that were easily distinguishable from the profile of sCJDMV1 (Fig. 1). GSS^F198S^ and GSS^A117V^ displayed low mw bands of ~ 8 and ~ 7 kDa as well as two broad bands comprised between 17-20 23–24 kDa for GSS^F198S^, and 16–18 and 22–23 kDa for GSS^A117V^; additional bands and smears of higher mw were also present more prominently in GSS^F198S^ (Fig. 1). By contrast, resPrP^D^ profile in sCJDMV1 included the typical three glycoform bands of ~ 30, ~ 27, and ~ 21 kDa of resPrP^D^ type 1. Two additional unique characteristics further distinguished GSS^A117V^ and GSS^F198S^ from sCJDMV1. Virtually all resPrP^D^ bands associated with these two GSS conditions were readily detected only with Abs to epitopes within the PrP ~ 51–150 region, but not with Abs recognizing more C-terminal epitopes (Fig. 1, panels b-f). Furthermore, GSS^A117V^ and GSS^F198S^ resPrP^D^ (as well as resPrP^D^ from sCJDMV1) showed no immunoreactivity with 8B4 Ab that recognizes the N-terminal epitope 36–43 (Fig. 1, panel a). This finding excludes the presence of full length resPrP^D^ in these two GSS variants, and is at variance with the presence of full-length resPrP^D^ reported in GSS^H187R^ [12]. In addition to epitope mapping, a second major distinguishing feature was that, in contrast to sCJDMV1 resPrP^D^, deglycosylation had no significant effect on the electrophoretic mobility of any of the major resPrP^D^ bands observed in GSS^F198S^ and GSS^A117V^, strongly suggesting that resPrP^D^ populating these bands is not glycosylated (Fig. 1). In a separate experiment carried out in GSS^F198S^, we also observed that the resPrP^D^ profile did not change after pretreatment of samples with strong denaturants such as guanidine hydrochloride and urea (Additional file 1: Figure S1). Collectively, these data suggest that at least two hmw bands seen in immunoblots of GSS^A117V^ and GSS^F198S^ resPrP^D^ represent covalently-linked multimers of the ~ 7 and ~ 8 kDa fragments (Fig. 1 and Additional file 1: Figure S1).

To definitely assess whether the higher molecular bands represented multimers (likely covalently-linked) of the ~ 7 and ~ 8 kDa internal fragments, as our epitope mapping and glycosylation study strongly suggested, we performed trypsin in-gel digestion of protein fragments on individual electrophoretic bands of purified resPrP^D^ from GSS^A117V^ and GSS^F198S^ (~ 7, 16–18 and 22–23 kDa in the case of GSS^A117V^ and ~ 8, 17–20 and 23–24 kDa in the case of GSS^F198S^) and did amino acid sequencing using mass spectrometry (Nano LC-MS).

**Fig. 2:**
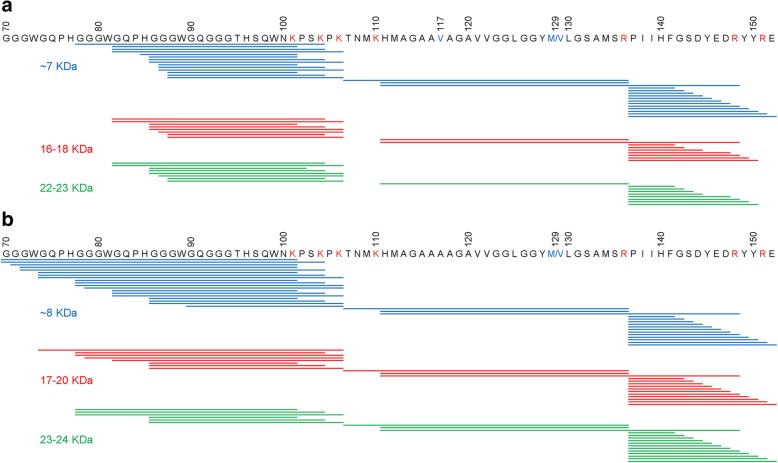
Mass spectrometry (MS)-based sequencing of PrP fragments in the material extracted from individual gel bands of two GSS variants. GSS^A117V^ resPrP^D^ (**a**) and GSS^F198S^ resPrP^D^ (**b**). The material present in these bands was subjected to trypsin digestion, followed by MS analysis of tryptic fragments. The fragments identified by MS for species extracted from individual gel bands are shown as blue, red and green lines. Amino acid sequence within the 70-152 region is shown above the lines. Residues marked in red represent potential cleavage sites in the relevant region of PrP; 129M/V polymorphic residues are marked in blue

The tryptic digest of the ~ 7 kDa resPrP^D^ fragment extracted from GSS^A117V^ and analyzed by Nano LC-MS revealed the presence of peptides exclusively from the central region of PrP between residues 78 and 152, with no detectable fragments from other parts of the protein. Trypsin is known to cleave polypeptide chains at the carboxyl side of lysine (K) or arginine (R). As shown in Fig. 2, seven potential trypsin cleavage sites are available within the central region of PrP between residues 70 and 153. Nano LC-MS analysis of the GSS^A117V^ ~ 7 kDa fragment using the Mascot Deamon searching engine identified fourteen trypsin-generated peptides with N-termini before the first potential trypsin cleavage site, two internal tryptic fragments, and twelve trypsin-generated peptides with N-terminus at the cleavage site following R136 (Fig. 2a and Additional file 2: Table S1). Altogether, these data demonstrated that in GSS^A117V^ the ~ 7 kDa fragment encompassed residues within the 78–152 region and had ragged N- and C-termini corresponding to residues 78/82/85–88 and 141–152, respectively. This is generally consistent with previous MALDI-derived data from extracts of PrP amyloid plaque cores, even though our Nano LC-MS analysis (which is more accurate) revealed that the N-terminus of this fragment may extend as far as up to residue 78 versus residue 85 previously reported based on the MALDI analysis [33].

A similar sequencing analysis of the 16–18 kDa and 22–23 kDa bands revealed the presence of essentially identical tryptic fragments, with the exception that the most N-terminal fragment started at residue 82, and the most C-terminal fragment ended at residue 150 (Fig. 2a). Remarkably, we could not detect any tryptic peptides from the region C-terminal to residue 150. Given that numerous peptides from the latter region are readily detectable by MS upon proteolytic digestion of resPrP^D^ from sCJD cases and mouse prion strains [1, 30], one can definitely conclude from these data that the 16–18 and 22–23 kDa species in GSS^A117V^ resPrP^D^ indeed represent oligomers (likely trimers and tetramers, respectively) of internal fragments encompassing residues within the 82–150 region. It is of note that fragments in the oligomers are somewhat shorter compared to those in the ~ 7 kDa band.

MS-based sequencing analysis of protein present in the resPrP^D^ ~ 8 kDa band from GSS^F198S^ demonstrated that this band contained rugged internal resPrP^D^ fragments from the 70–152 region, with N-termini between residues 70 and 90 and C-termini between residues 141 and 152 (Fig. 2b and Additional file 2: Table S2). Again, MS analysis did not reveal the presence of peptides from parts of PrP other than the central region between residues 70 and 152. Previous sequencing studies of the ~ 8 kDa fragment (or fragments of similar kDa) extracted from PrP amyloid plaques and analyzed by Edman degradation chemistry alone or combined with automated sequencing identified the major N-terminus at residues G58, G74 and G81 while the C-terminus was reported at residue 150 [27, 34, 35].

Very similar internal fragments were identified in higher molecular bands of 17–20 and 23–24 kDa in GSS^F198S^ resPrP^D^, with the exception that the longest of these fragments had N-termini at residue 74 and 78 in the 17–20 and 23–24 kDa bands, respectively (Fig. 2b). Importantly, akin to the finding for GSS^A117V^ resPrP^D^, no peptides from the region C-terminal to residue 152 were detected by MS in tryptic digests of protein in these two higher molecular weight bands. Thus, also in GSS^F198S^ resPrP^D^, the latter bands contained covalently-linked oligomers of the internal PrP fragments within the 74–78/142–152 region, which, as in GSS^A117V^, are somewhat shorter than the ~ 8 kDa monomer.

**Fig. 3:**
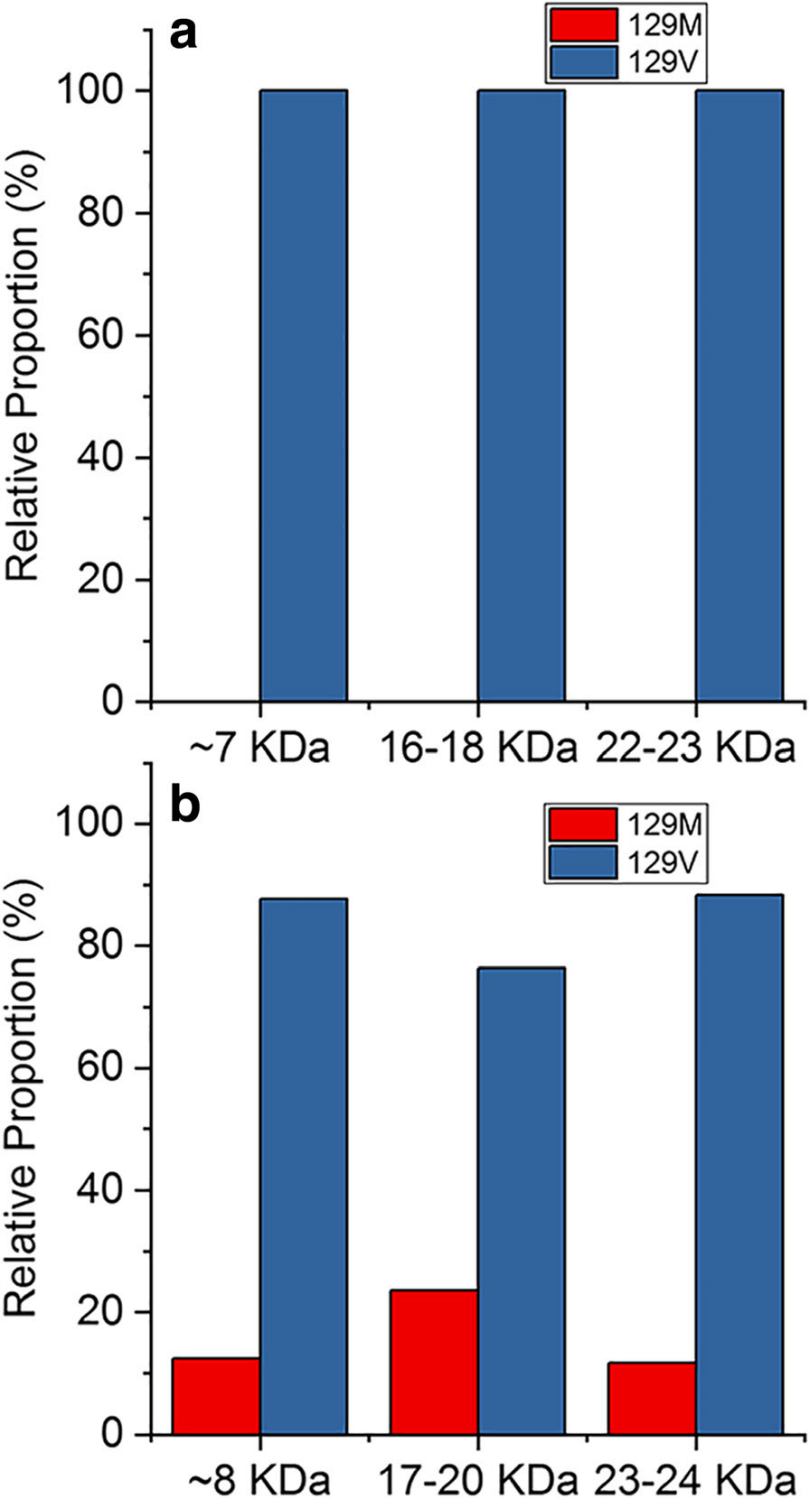
Relative abundance of 129M and 129V PrP variants in resPrP^D^ associated with GSS^A117V^ and GSS^F198S^. **a**: GSS^A117V^; **b**: GSS^F198S^. The relative abundance of resPrP^D^ with M or V at residue 129 reflects the representation of the PrP mutation, which is coupled with the 129V in both GSS variants. Approximately 10–25% of resPrP^D^ could be identified as non-mutated (wild type) in GSS^F198S^ while only mutated resPrP^D^ could be detected in GSS^A117V^. The relative populations were determined by mass spectrometry using the spectral counting method

Using Nano LC-MS we also determined the relative representation in resPrP^D^ of the 129M and 129V polymorphic forms of the prion protein. Consistent with a previous report, resPrP^D^ from GSS^A117V^ was invariably 100% 129V, with no detectable 129M polymorph [33]. By contrast, and at variance with a previous report, in GSS^F198S^ resPrP^D^, all three bands examined consistently showed the presence of both polymorphic variants, with a large dominance (76–88%) of the 129V polymorph (Fig. 3) [35].

## Discussion

The present results indicate that the mechanism of resPrP^D^ aggregation in GSS^A117V^ and GSS^F198S^ involves formation of covalently-linked multimers of the ~7-8 kDa internal fragments. Furthermore, it has been previously shown by Edman degradation chemistry that the 7 and 14 kDa fragments in GSS^H187R^ resPrP^D^ share the N-terminus, suggesting that formation of covalently-linked multimers may also take place in the latter GSS variant [12]. PrP bands suggestive of dimerization have been reported in cell systems and brain tissues [13, 29]. However, the resistance to PK digestion of these PrP species and, more importantly, their molecular nature have not been determined. In this context, the present data provide the strongest evidence to date for the presence of covalently cross-linked species of resPrP^D^ in prion diseases, suggesting that these previously unrecognized species may play a major role in the pathogenesis of human prion diseases. Of note, covalent crosslinking has been reported for toxic aggregates of α-synuclein and Aβ involved in Parkinson’s and Alzheimer’s diseases, respectively [2, 3, 9, 10, 32].

This novel finding has important implications for understanding phenotypic variability in human prion diseases. Even though resPrP^D^ aggregates associated with both CJD and GSS phenotypes have been shown to be transmissible, the dramatic difference between these species suggests fundamentally different structural mechanisms of prion protein conformational conversion in GSS as compared with those in CJD [6, 28]. Indeed, the absence of the constraints imposed by glycans and the GPI anchor (that are missing in ~ 7–8 kDa fragments and their covalently-linked multimers) is likely to allow for different types of resPrP^D^ assemblies. A potential structural model for the ~ 7–8 kDa fragments and their multimers is provided by synthetic amyloid fibrils generated from PrP23–144, a protein matching the sequence of the C-terminally truncated PrP harbored in GSS^Y145Stop^ [11, 17]. These synthetic amyloid fibrils adopt a parallel in-register β-structure [36] that is fundamentally different from the 4-rung solenoid model proposed for the CJD-associated resPrP^D^ [5, 37]. Remarkably, diseased mice inoculated with PrP23–144 fibrils accumulate GSS-like resPrP^D^ aggregates (consisting of ~ 7 kDa fragment) that likely share the structural motif of amyloid fibrils used as the inoculum [11].

The nature of the covalent crosslinks between PrP fragments and the residues involved in these crosslinks are at present unknown. However, our data strongly suggest that at least two different types of linkages (between different residues) are likely involved in coupling the monomers that form trimers and tetramers, since a single linkage type would preclude mass spectrometric identification of all tryptic fragments from the ~ 80–150 region.

Although the basic structural characteristics and mode of aggregation of resPrP^D^ in GSS^A117V^ and GSS^F198S^ appear to be similar, at least two features distinguish these two conditions. First, substantially more resPrP^D^ populates the hmw regions of the immunoblot in GSS^F198S^ than in GSS^A117V^, suggesting a stronger propensity for covalent polymerization in GSS^F198S^. Second, resPrP^D^ associated with GSS^A117V^ consists solely of fragments containing the 129V polymorphic form of PrP, whereas both polymorphs are present in GSS^F198S^, even though the 129V form predominates. Since pathogenic mutations in both GSS variants are on the background of 129V PrP, this implies that resPrP^D^ in GSS^F198S^ contains internal PrP fragments derived both from both the mutant as well as wild-type proteins. By contrast, the internal PrP fragments populating GSS^A117V^ resPrP^D^ represent exclusively the mutant protein. This suggests that the A117V mutation (which is within the PK-resistant fragments) may impede the templated conversion of wild type PrP. No such impediment would be expected in GSS^F198S^ where the pathogenic mutation is outside the PK-resistant region.

Recently, a novel eight-residue insertion in the hydrophobic region of PrP has been associated with a GSS phenotype [23]. Brains of transgenic mice harboring the corresponding mouse insertion variation revealed the presence of a 8 kDa resPrP^D^ fragment similar to those observed in other GSS variants as well as a 16 kDa thermolysin-resistant fragment mapping to residues ~ 23–155. Based on this finding and other data, it was suggested that the 8 kDa internal fragment derived from the N-terminal truncation of the 16 kDa fragment and that this mechanism was shared by other GSS variants. While this hypothesis may apply to the mouse model and, possibly, to this specific GSS insertion variant, our demonstration that hmw bands harbor oligomers of the ~ 7 and ~ 8 kDa fragments in GSS^A117V^ and GSS^F198S^ (and possibly GSS^H187R^) makes the proposed mechanism not applicable to these GSS variants.

## Conclusion

We demonstrated for the first time that the formation of multimers of a small internal fragment is the primary mechanism of prion aggregation in GSS^A117V^ and GSS^F198S^, two classical variants of Gerstmann-Sträussler-Scheinker disease (GSS). Prion aggregation by covalently-linked multimer formation from a small glycan- and GPI-free fragment is likely to allow for novel prion assembles. This finding opens new horizons and likely will stimulate new research. Covalently-linked multimers formation has been reported in other neurodegenerative diseases such as Parkinson’s and Alzheimer’s diseases.

## Additional files

Additional file 1: **Figure S1.** Further biochemical characterization of resPrP^D^ associated with GSS^F198S^. Lane 1: PNGase F-deglycosylated resPrP^D^ from brain homogenate immunoblotted with 3F4 following standard conditions. Lane 2: with additional boiling, freezing-thawing and sonication pre-deglycosylation; lane 3: 41 h PNGase F treatment; lanes 4–7: incubation with strong denaturants, 8 M urea (after ethanol or methanol precipitation, lanes 4, 5) and 8 M guanidine hydrochloride at 80 °C (after ethanol or methanol precipitation, lanes 6, 7) pre-deglycosylation. Treatments had no detectable effect on the resPrP^D^ electrophoretic profile. (TIF 17802 kb)

Additional file 2: **Table S1.** Tryptic peptides identified for ~ 7 KDa band in GSS^A117V^ resPrP^D^. Residue at position 129 is marked in blue. **Table S2.** Tryptic peptides identified for ~ 8 KDa band in GSS^F198S^ resPrP^D^. (PDF 122 kb)

## Abbreviations

Ab: Antibody
BH: Brain homogenates
CJD: Creutzfeldt-Jakob disease
GPI: Glycosylphosphatidylinositol
GSS: Gerstmann-Sträussler-Scheinker disease
hmw: Higher molecular weight
K: Lysine
kDa: Kilodaltons
M: Methionine
MS: Mass spectrometry
mw: Molecular weight
Nano LC-MS/MS: Nano liquid chromatography mass spectrometry
NPDPSC: National Prion Disease Pathology Surveillance Center
PK: Proteinase K
PMSF: Phenylmethanesulfonyl fluoride
PrP^D^: Disease-related prion protein
PVDF: Polyvinylidene difluoride
R: Arginine
resPrP^D^: Disease-related proteinase K-resistant prion protein
SDS: Sodium dodecyl sulfate
TBS: Tris buffered saline
V: Valine
