# Supplementary figures and images for: Gerstmann-Sträussler-Scheinker disease revisited: accumulation of covalently-linked multimers of internal prion protein fragments
Source: Acta Neuropathol Commun. 2019 May 29;7:1. doi: 10.1186/s40478-019-0734-2 (PMC6540574; doi:10.1186/s40478-019-0734-2)

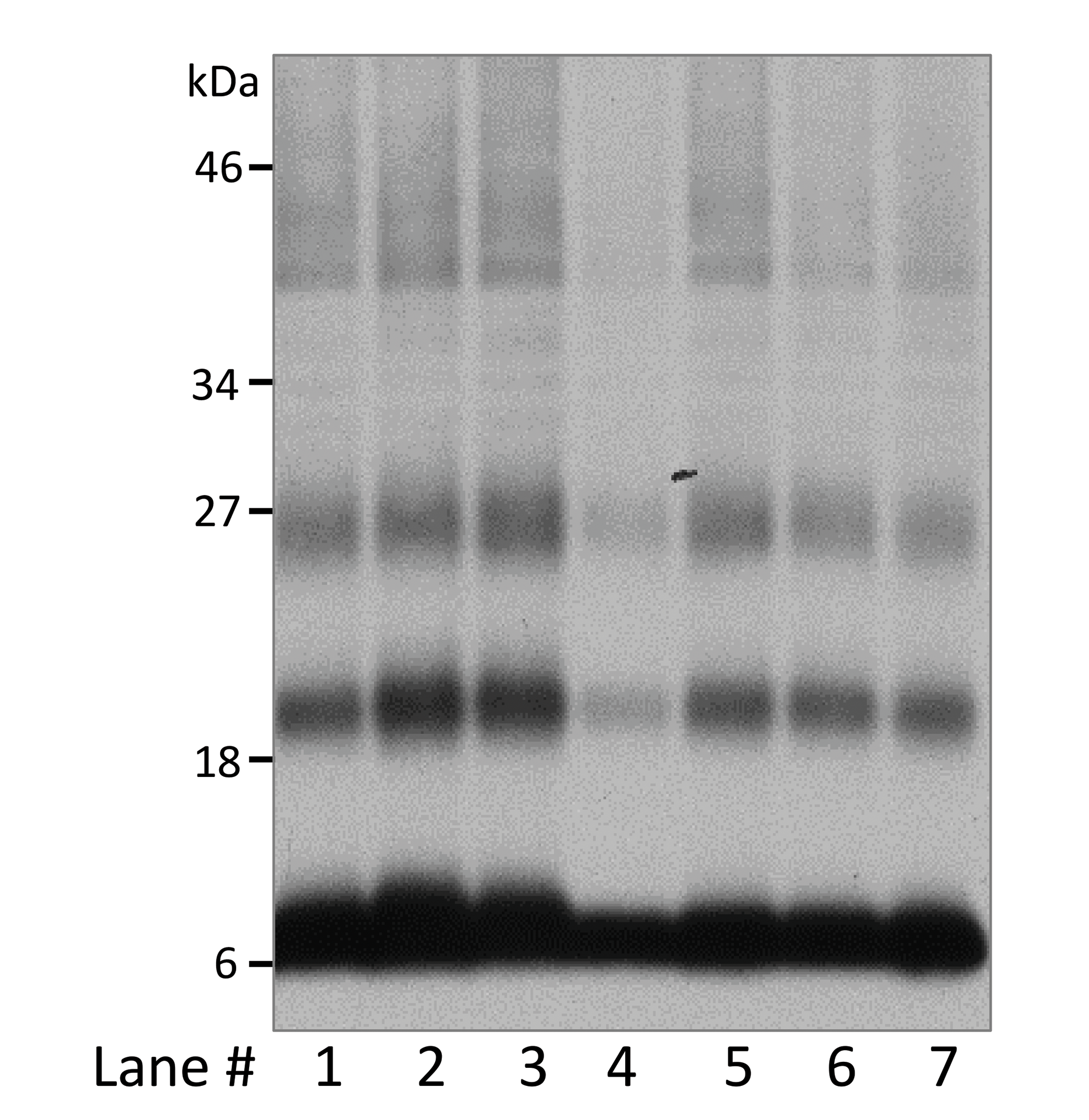

Supplement: Supplementary file 1 — Figure S1. Further biochemical characterization of resPrPD associated with GSSF198S. Lane 1: PNGase F-deglycosylated resPrPD from brain homogenate immunoblotted with 3F4 following standard conditions. Lane 2: with additional boiling, freezing-thawing and sonication pre-deglycosylation; lane 3: 41 h PNGase F treatment; lanes 4–7: incubation with strong denaturants, 8 M urea (after ethanol or methanol precipitation, lanes 4, 5) and 8 M guanidine hydrochloride at 80 °C (after ethanol or methanol precipitation, lanes 6, 7) pre-deglycosylation. Treatments had no detectable effect on the resPrPD electrophoretic profile. (TIF 17802 kb) [file 40478_2019_734_MOESM1_ESM.tif]
