# Supplementary material for: Gerstmann-Sträussler-Scheinker disease revisited: accumulation of covalently-linked multimers of internal prion protein fragments
Source: Acta Neuropathol Commun. 2019 May 29;7:1. doi: 10.1186/s40478-019-0734-2 (PMC6540574; doi:10.1186/s40478-019-0734-2)
Supplement: Supplementary file 2 — Table S1. Tryptic peptides identified for ~ 7 KDa band in GSSA117V resPrPD. Residue at position 129 is marked in blue. Table S2. Tryptic peptides identified for ~ 8 KDa band in GSSF198S resPrPD. (PDF 122 kb) [file 40478_2019_734_MOESM2_ESM.pdf]

Gerstmann-Sträussler-Scheinker disease revisited: Accumulation of covalently-linked multimers of internal prion protein fragments

Laura Cracco[1], Xiangzhu Xiao[2,*], Satish K. Nemani[1,*], Jody Lavrich[1], Ignazio Cali[1,3], Bernardino Ghetti[4], Silvio Notari[1], Witold K. Surewicz[2], Pierluigi Gambetti[1,✉]

[1]Department of Pathology, Case Western Reserve University, Cleveland, OH, USA.
[2]Department of Physiology and Biophysics, Case Western Reserve University, Cleveland, OH, USA.
[3]National Prion Disease Pathology Surveillance Center, Case Western Reserve University, Cleveland, OH, USA.
[4]Department of Pathology and Laboratory Medicine, Indiana University School of Medicine, Indianapolis, IN, USA.

*The two authors have equally contributed to the study

✉ Corresponding author: Pierluigi Gambetti, Department of Pathology, Case Western Reserve University, 2085 Adelbert Road, Room 419, Cleveland, OH 44106, USA. Phone: +1-216-410-6883, email: pxg13@case.edu.

Supplementary Material

Figure S1. Further biochemical characterization of resPrP^{D} associated with GSS^{F198S}. Lane 1: PNGase F-deglycosylated resPrP^{D} from brain homogenate immunoblotted with 3F4 following standard conditions. Lane 2: with additional boiling, freezing-thawing and sonication pre-deglycosylation; lane 3: 41h PNGase F treatment; lanes 4-7: incubation with strong denaturants, 8M urea (after ethanol or methanol precipitation, lanes 4, 5) and 8M guanidine hydrochloride at 80° C (after ethanol or methanol precipitation, lanes 6, 7) pre-deglycosylation. Treatments had no detectable effect on the resPrP^{D} electrophoretic profile.

Table S1. Tryptic peptides identified for ~7 KDa band in GSS^{A117V} $resPrP^{D}$. Residue at position 129 is marked in blue.

	Peptide	129M/V	Sequence	Mass (calculated)	Mass (experimental)	Note
N-terminal peptides	78-104		GGGWGQPHGGGWGQGGGTHSQWNKPSK	2701.2283	2701.2345	
	82-101		GQPHGGGWGQGGGTHSQWNK	2031.9049	2031.9058	
	82-104		GQPHGGGWGQGGGTHSQWNKPSK	2344.0846	2344.0849	
	82-106		GQPHGGGWGQGGGTHSQWNKPSKPK	2569.2323	2569.2341	
	85-101		HGGGWGQGGGTHSQWNK	1749.7720	1749.7733	
	86-101		GGGWGQGGGTHSQWNK	1612.7131	1612.7140	
	86-104		GGGWGQGGGTHSQWNKPSK	1924.8929	1924.8963	
	86-106		GGGWGQGGGTHSQWNKPSKPK	2150.0406	2150.0411	
	87-101		GGWGQGGGTHSQWNK	1555.6917	1555.6936	
	87-104		GGWGQGGGTHSQWNKPSK	1867.8714	1867.8729	
	87-106		GGWGQGGGTHSQWNKPSKPK	2093.0191	2093.0192	
	88-101		GWGQGGGTHSQWNK	1498.6702	1498.6710	
	88-104		GWGQGGGTHSQWNKPSK	1810.8499	1810.8506	
	88-106		GWGQGGGTHSQWNKPSKPK	2035.9977	2035.9983	
middle peptides	107-136	129V	K.TNMKHMAGAAVAGAVVGGLGGYVLGSAMSR	2880.4045	2880.4054	Oxidation of 3 methionines
	111-136		K.HMAGAAVAGAVVGGLGGYVLGSAMSR	2358.1937	2358.1937	
	111-136		K.HMAGAAVAGAVVGGLGGYVLGSAMSR	2374.1886	2374.1888	Oxidation of 1 methionine
	111-136		K.HMAGAAVAGAVVGGLGGYVLGSAMSR	2390.1835	2390.1837	Oxidation of 2 methionines
	111-148		K.HMAGAAVAGAVVGGLGGYVLGSAMSRPIIHFGSDYEDR	3787.8563	3787.8571	
	111-148		K.HMAGAAVAGAVVGGLGGYVLGSAMSRPIIHFGSDYEDR	3803.8512	3803.8515	Oxidation of 1 methionine
	111-148		K.HMAGAAVAGAVVGGLGGYVLGSAMSRPIIHFGSDYEDR	3819.8461	3819.8461	Oxidation of 2 methionines
C-terminal peptides	137-141		R.PIIHF	625.3588	625.3589	
	137-142		R.PIIHFG	682.3802	682.3806	
	137-143		R.PIIHFGS	769.4123	769.4137	
	137-144		R.PIIHFGSD	884.4392	884.4391	
	137-145		R.PIIHFGSDY	1047.5025	1047.5040	
	137-146		R.PIIHFGSDYE	1176.5451	1176.5510	
	137-147		R.PIIHFGSDYED	1291.5721	1291.5727	
	137-148		R.PIIHFGSDYEDR	1447.6732	1447.6734	
	137-149		R.PIIHFGSDYEDRY	1610.7365	1610.7376	
	137-150		R.PIIHFGSDYEDRYY	1773.7998	1773.8003	
	137-151		R.PIIHFGSDYEDRYYR	1929.9009	1929.9052	
	137-152		R.PIIHFGSDYEDRYYRE	2058.9435	2059.9217	

Table S2. Tryptic peptides identified for ~8 KDa band in GSS^{F198S} resPrP^{D}.

	Peptide	129M/V	Sequence	Mass (calculated)	Mass (experimental)	Note
N-terminal peptides	70-101		GGGWGQPHGGGWGQPHGGGWGQGGGTHSQWNK	3165.3840	3165.3854	
	70-104		GGGWGQPHGGGWGQPHGGGWGQGGGTHSQWNKPSK	3477.5638	3477.5649	
	71-101		GGWGQPHGGGWGQPHGGGWGQGGGTHSQWNK	3108.3626	3108.3630	
	72-101		GWGQPHGGGWGQPHGGGWGQGGGTHSQWNK	3051.3411	3051.3403	
	72-104		GWGQPHGGGWGQPHGGGWGQGGGTHSQWNKPSK	3363.5208	3363.5251	
	74-101		GQPHGGGWGQPHGGGWGQGGGTHSQWNK	2808.2403	2808.2424	
	74-104		GQPHGGGWGQPHGGGWGQGGGTHSQWNKPSK	3120.4201	3120.4206	
	74-106		GQPHGGGWGQPHGGGWGQGGGTHSQWNKPSKPK	3345.5678	3345.5678	
	78-101		GGGWGQPHGGGWGQGGGTHSQWNK	2389.0486	2389.0450	
	78-104		GGGWGQPHGGGWGQGGGTHSQWNKPSK	2701.2283	2701.2325	
	78-106		GGGWGQPHGGGWGQGGGTHSQWNKPSKPK	2926.3761	2926.3754	
	79-106		GGWGQPHGGGWGQGGGTHSQWNKPSKPK	2869.3546	2869.3620	
	82-101		GQPHGGGWGQGGGTHSQWNK	2031.9049	2031.9092	
	82-104		GQPHGGGWGQGGGTHSQWNKPSK	2344.0846	2344.0936	
	82-106		GQPHGGGWGQGGGTHSQWNKPSKPK	2569.2323	2569.2341	
	86-101		GGGWGQGGGTHSQWNK	1612.7131	1612.7177	
	86-104		GGGWGQGGGTHSQWNKPSK	1924.8929	1924.8946	
	86-106		GGGWGQGGGTHSQWNKPSKPK	2150.0406	2150.0407	
	90-106		GQGGGTHSQWNKPSKPK	1792.8969	1792.9002	
middle peptides	107-136	129M	K.TNMKHMAGAAAAGAVVGGLGGYMLGSAMSR	2852.3554	2852.3681	Oxidation of 1 methionine
	111-136		K.HMAGAAAAGAVVGGLGGYMLGSAMSR	2378.1293	2378.1236	Oxidation of 1 methionine
	111-136		K.HMAGAAAAGAVVGGLGGYMLGSAMSR	2394.1243	2394.1223	Oxidation of 2 methionines
	111-136		K.HMAGAAAAGAVVGGLGGYMLGSAMSR	2410.1192	2410.1186	Oxidation of 3 methionines
	107-136	129V	K.TNMKHMAGAAAAGAVVGGLGGYVLGSAMSR	2836.3782	2836.3754	Oxidation of 2 methionines
	107-136		K.TNMKHMAGAAAAGAVVGGLGGYVLGSAMSR	2852.3732	2852.3735	Oxidation of 3 methionines
	111-136		K.HMAGAAAAGAVVGGLGGYVLGSAMSR	2330.1624	2330.1630	
	111-136		K.HMAGAAAAGAVVGGLGGYVLGSAMSR	2346.1573	2346.1578	Oxidation of 1 methionine
	111-136		K.HMAGAAAAGAVVGGLGGYVLGSAMSR	2362.1522	2362.1532	Oxidation of 2 methionines
	111-148		K.HMAGAAAAGAVVGGLGGYVLGSAMSRPIIHFGSDYEDR	3775.8199	3776.8312	Oxidation of 1 methionine
	111-148		K.HMAGAAAAGAVVGGLGGYVLGSAMSRPIIHFGSDYEDR	3791.8184	3791.8163	Oxidation of 2 methionines
C-terminal peptides	137-141		R.PIIHF	625.3588	625.3590	
	137-142		R.PIIHFG	682.3802	682.3802	
	137-143		R.PIIHFGS	769.4123	769.4120	
	137-144		R.PIIHFGSD	884.4392	884.4397	
	137-145		R.PIIHFGSDY	1047.5025	1047.5023	
	137-146		R.PIIHFGSDYE	1176.5451	1176.5479	
	137-147		R.PIIHFGSDYED	1291.5721	1291.5721	
	137-148		R.PIIHFGSDYEDR	1447.6732	1447.6731	
	137-149		R.PIIHFGSDYEDRY	1610.7365	1061.7372	
	137-150		R.PIIHFGSDYEDRYY	1773.7998	1773.8005	
	137-151		R.PIIHFGSDYEDRYYR	1929.9009	1930.8829	
	137-152		R.PIIHFGSDYEDRYYRE	2058.9435	2059.9249	
